# Dual Enzyme-Triggered Controlled Release on Capped Nanometric Silica Mesoporous Supports

**DOI:** 10.1002/open.201200003

**Published:** 2012-02-17

**Authors:** Alessandro Agostini, Laura Mondragón, Carmen Coll, Elena Aznar, M Dolores Marcos, Ramón Martínez-Máñez, Félix Sancenón, Juan Soto, Enrique Pérez-Payá, Pedro Amorós

**Affiliations:** [a] Centro de Reconocimiento Molecular y Desarrollo Tecnológico, Unidad Mixta Universidad Politécnica de Valencia, Universidad de Valencia (Spain); [b] Departamento de Química, Universidad Politécnica de ValenciaCamino de Vera s/n, 46022, Valencia (Spain) E-mail: rmaez@qim.upv.es; [c] CIBER de Bioingeniería, Biomateriales y Nanotecnología (CIBER-BNN, Spain); [d] Centro de Investigación Príncipe Felipe, Laboratorio de Péptidos y ProteínasAvda. Autopista al Saler, 16, 46012, Valencia (Spain); [e] Institut de Ciència dels Materials (ICMUV), Universitat de ValenciaP. O. Box 2085, 46071, Valencia (Spain)

**Keywords:** controlled release, drug delivery, mesoporous materials, nanoparticles, urease

The development of nanoscopic hybrid materials equipped with “molecular gates” showing the ability of releasing target entrapped guests upon the application of an external trigger has attracted great attention and has been extensively explored during recent years.^1^ These nanodevices are composed of two subunits, namely, a suitable support and certain capping entities grafted on the surface of the scaffolding.^2^ The support is used as a suitable reservoir in which certain chemicals can be stored whereas the molecules grafted in the outer surface act as a “gate” and can control the release of the entrapped molecules at will. Both components are carefully selected and arranged in order to achieve a wide range of required functionalities.

As support, mesoporous silica nanoparticles (MSN) have been widely used due to their unique properties, such as large load capacity, biocompatibility, high surface area and well-known functionalization procedures.^3^ Moreover, gated MSN have recently been used for the development of on-command delivery nanodevices by using several physical and chemical triggers. For instance, MSN displaying controlled-release features with the use of light,^4^ redox reactions,^5^ and pH changes^6^ have been described. In contrast, gated nanomaterials able to deliver the cargo triggered by biomolecules are scarce although some illustrative examples that use antigen–antibody interactions,^7^ hybridisation of single stranded oligonucleotides,^8^ and enzymes^9^–^14^ have been reported. In particular, the use of enzymes is especially appealing taking into account the possibility to synthesise tailor-made enzyme-specific sequences as molecular caps. However, in spite of these interesting features there are few examples that use enzymes in opening protocols. The first enzyme-responsive gate in a mesoporous support was described by Stoddart and co-workers. In that work, a [2]rotaxane ended with a bulky adamantyl ester stopper that acted as molecular gate was removed by porcine liver esterase treatment.^9^ Further hybrid systems involving avidin–biotin,^10^ lactose,^11^ starch,^12^ β-cyclodextrins^13^ and peptide sequences^14^ as capping groups have been reported. These examples offer a chemically simple approach that can benefit from the vast knowledge on enzyme–substrate pairs for the design of versatile systems for controlled release.

A further step in the field should take into account that the flow of defined biological processes rely on biochemical networks with the participation of multiple enzyme-dependent stages. It would be then useful to define future applications with the development of dual or multiple enzyme-triggered systems by using capped mesoporous supports. This would require the design of capping threads containing different enzyme-specific hydrolysable linkers located at defined positions on the external surface of MSN. The whole design will provide highly versatile and specific-release nanodevices the delivery profiles of which could be controlled and fine-tuned by defined combinations of enzymes. As a first-of-its-kind proof-of-concept, we have prepared an MSN support capped with the molecular entity **1** that contains amide and urea linkages, and we have evaluated it as a multi-enzyme-tuned delivery system (Scheme 1).

**Scheme 1:**
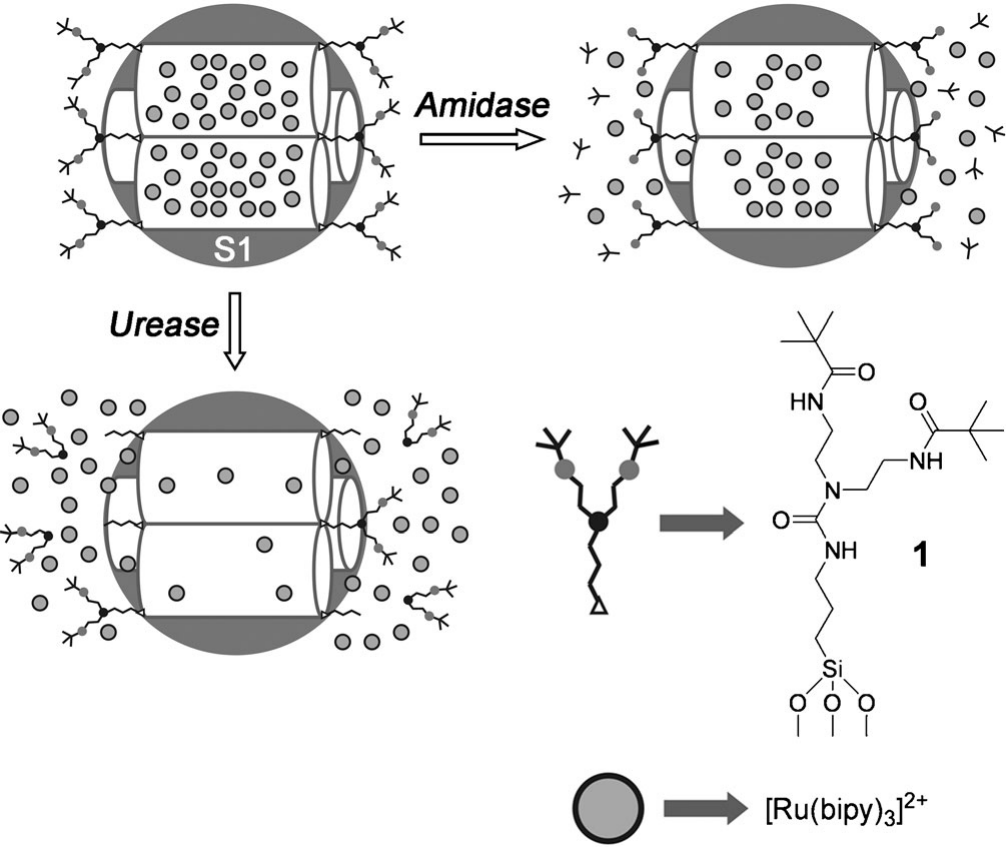
Schematic representation of solid S1 and the enzymatic uncapping mechanism.

As inorganic carrier vehicle we selected mesoporous MCM-41 silica nanoparticles of about 100 nm in diameter, which were prepared following well-known procedures using TEOS as hydrolytic inorganic precursor and hexadecyltrimethylammonium bromide (CTABr) as porogen species.^15^ The structure of the nanoparticulated calcined MCM-41 starting material was confirmed by X-ray diffraction (Figure 1), TEM and SEM microscopy. The N_2_ adsorption–desorption isotherms showed a typical type *IV* curve with a specific surface of 999.6 m^2^ g^−1^, and a pore volume of 0.79 cm^3^ g^−1^. From the XRD, porosimetry and TEM studies, the *a*_0_ cell parameter (4.44 nm), the pore diameter (2.46 nm) and a value for the wall thickness (1.99 nm) were calculated. For the preparation of **S1**, the calcinated MSN was first loaded with [Ru(bipy)_3_]Cl_2_, which was used as dye for monitoring the enzyme-triggered protocol, and then treated with the capping molecule **1**. The derivative **1** was synthesised following a two-step procedure from diethylentriamine. In a first step both primary amine moieties of diethylentriamine were selectively amidated with pivaloyl anhydride at 0 °C.^16^ In a second step, the free secondary amine was treated with (3-isocyanatopropyl)triethoxysilane in the presence of K_2_CO_3_ to yield the final trialcoxysilane derivative **1** (see the Supporting Information for experimental details).

**Figure 1:**
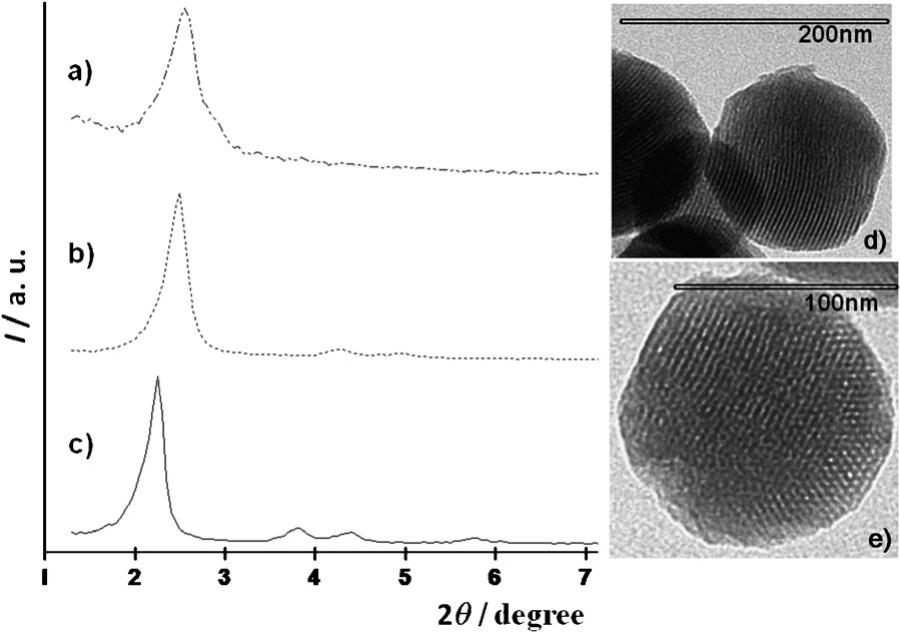
Left: powder X-ray patterns of: a) solid S1, b) calcined MCM-41, and c) MCM-41 as-synthesised. Right: TEM images of: d) solid S1, and e) calcined MCM-41 sample showing the typical hexagonal porosity of the MCM-41 mesoporous matrix.

The prepared solid **S1** was characterised by using standard techniques. **S1** displays expected features of the MCM-41 phase, as can be observed in the TEM image shown in Figure 1. This suggests that loading and grafting procedures did not modify the mesoporous structure of the starting material. Additionally the N_2_ adsorption–desorption isotherm of **S1** was typical of mesoporous systems with filled mesopores, and a significant decrease in the N_2_ volume adsorbed and the surface area (696.5 m^2^ g^−1^) was observed. The content of ruthenium complex and capping molecule **1** in the final solid **S1** were determined by thermogravimetric and elemental analysis and amounted to 0.15 and 0.22 mmol g^−1^ SiO_2_, respectively.

The presence of amide and urea moieties in the capping molecule **1** allowed the analysis of a multi-enzyme-dependent release of the [Ru(bipy)_3_]^2+^ dye from **S1** by using amidase and urease enzymes. In a typical experiment, solid **S1** (5 mg) was suspended in water (12.5 mL) at pH 7.5. Then amidase or/and urease (1 μL of the purchased solution) were added and the final suspension was stirred. As the control experiment dye release was determined by using suspensions of **S1** under similar conditions but in the absence of enzyme. Uncapping and subsequent delivery of the dye to the aqueous solution was easily detected by monitoring the metal-to-ligand charge transfer transition band of the [Ru(bipy)_3_]^2+^ dye at 451 nm or through the emission band at 619 nm (*λ*_ex_=451 nm).^17^ The different delivery profiles for both experiments are shown in Figure 2. In the presence of amidase a relatively quick delivery was found (ca. 20 % of the cargo was released in 2 h); however, only a moderate delivery was observed for long periods of time (only 40 % of the dye was delivered after 15 h). In contrast the urease-stimulated release was slower (for instance, no delivery was observed after 2.5 h) yet at longer time periods urease was able to deliver a significantly larger amount of the cargo from **S1** (ca. 80 % of the dye was delivered after 15 h) than amidase. We also observed that there is a very low payload release in the absence of urease or amidase (Figure 2).

**Figure 2:**
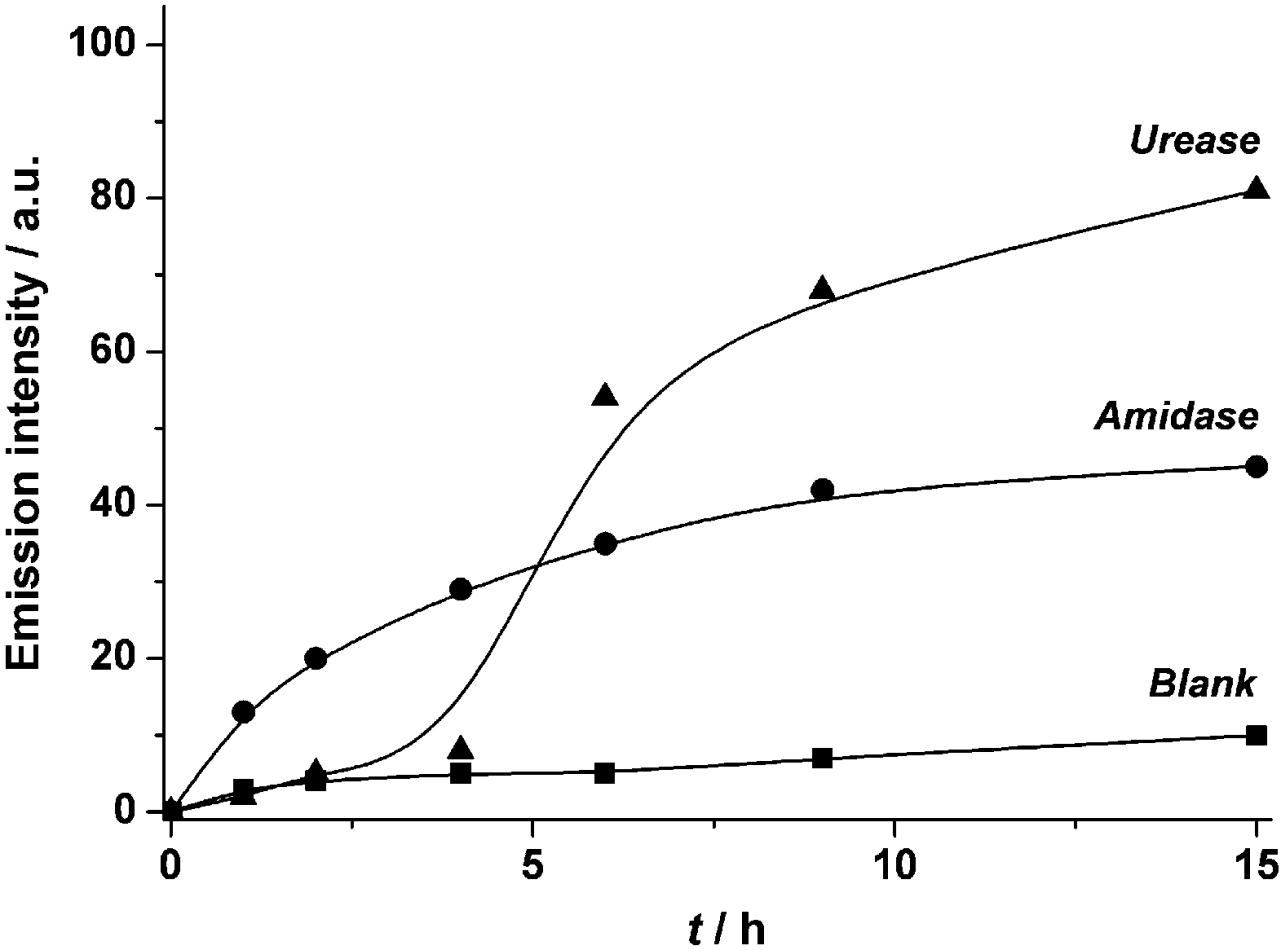
Kinetics of the release of [Ru(bipy)_3_]^2+^ dye from solid S1 in the presence of amidase and urease.

The different enzyme-dependent delivery rates obtained on solid **S1** were related to the design of gate **1**, in particular to the relative position of the hydrolysable groups on the molecule. Solid **S1** treatment with amidase induced the hydrolysis of the amide bonds located far away from the surface with the subsequent release of two bulky trimethylacetate moieties. In spite of this reduction in the steric crowding around the pore outlets, the organic residual that remained anchored was large enough to hamper, to some extent, the release of the dye. As a consequence, the degree of cargo release is low but fast. On changing from amidase to urease a completely different response was obtained. Addition of urease induced the hydrolysis of the urea bond located deeper inside the structure of **1** and led to a drastic reduction of the thread size. As a consequence, the degree of cargo release is high but quite slow.

Release experiments with **S1** in the presence of both enzymes were also carried out. In this case, a synergic effect was observed; the action of the amidase induced a rapid cargo release, whereas at longer time periods the combined action of the urease allows a nearly complete cargo delivery (see the Supporting Information). In order to further demonstrate that the enzyme treatment is entirely responsible for the cargo release, two additional experiments were carried out. Solid **S1** was incubated in the presence of non-related enzymes, such as esterase and pronase. In a different experiment, prior to incubation with solid **S1**, the amidase and urease were heat denatured at 60 °C for 60 min. In both experiments no release of the dye was observed.

Once we demonstrated the in vitro aperture mechanism of **S1**, our next objective was to test the feasibility of using this gated nanodispositive in cells. For this purpose, the HeLa cell line was chosen and treated with **S1** at different doses for 24 h. Cell viability and cellular uptake of nanoparticles was assessed by using the WST-1 assay and confocal microscopy (Figure 3).^12^ The same experiments were also performed by using the MCF-7 cell line (see the Supporting Information for further details). Confocal images demonstrated that the intracellular vesicular localization of **S1** nanoparticles (red) is probably associated to lysosomes. Also, **S1** solid was biocompatible at the concentrations tested as no significant reduction in cell viability was observed.

**Figure 3:**
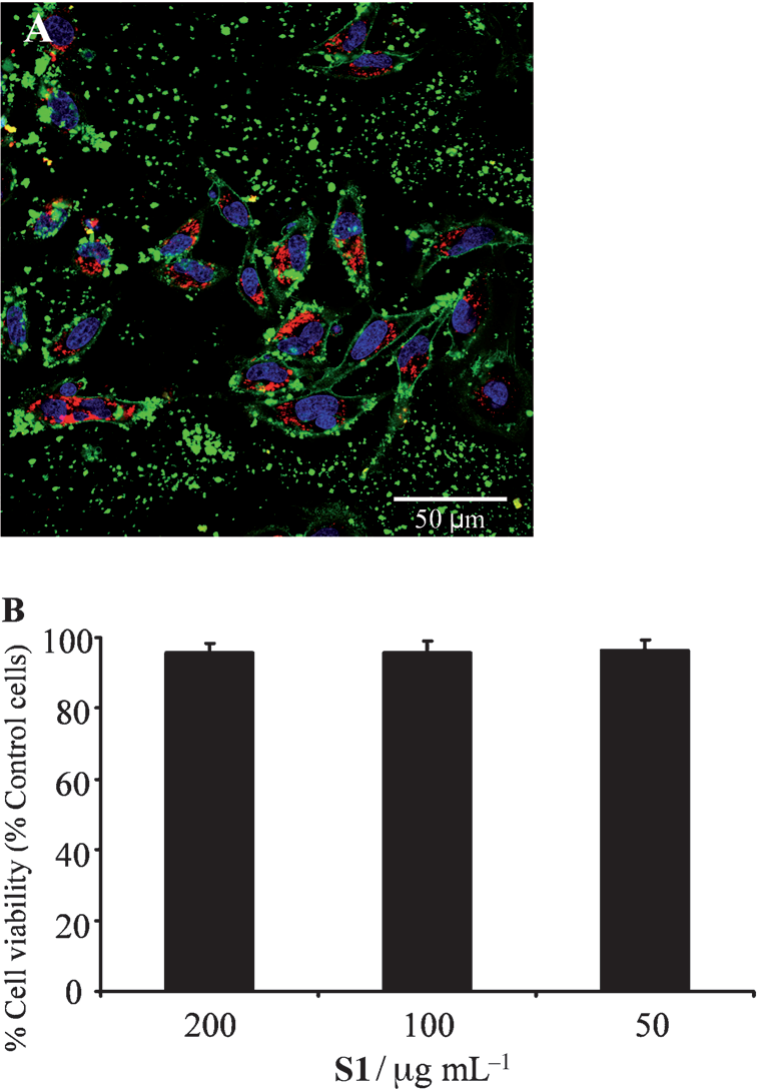
A) Confocal microscopy images corresponding to HeLa cells treated with solid S1 (100 μg mL^−1^). The cellular uptake of S1 was followed by [Ru(bipy)_3_]^2+^ associated fluorescence (red) in the presence of DNA marker Hoechst 33342 (blue) and the plasma membrane marker WGA-AlexaFluor 647 (green). B) For viability studies, cells were treated with S1 and after 24 h incubation, WST-1 reagent was added and cell viability was measured.

With the aim to demonstrate a possible therapeutic application of these nanodevices as drug carriers, a new **S1** solid was synthesised containing the chemotherapeutic agent camptothecin (CPT). CPT is a cytotoxic quinoline alkaloid that inhibits DNA polymerase I and disrupts DNA replication to induce cell death. HeLa cells were treated as described before in the presence of this new material, **S1**-CPT.

In order to obtain a more detailed analysis of the cell death processes related to the in-cell release of CPT from the nanoparticles, the dye propidium iodide (PI) and the early stage cell death marker Annexin V (Ann V) were employed (see the Supporting Information for further details). Figure 4 shows the results obtained by confocal microscopy and flow cytometry. A significant reduction in cell viability was observed 24 h after the addition of **S1**-CPT by confocal microscopy studies (cells detached from the plate, plasma membrane blebbling, and presence of cellular debris, among other features). These results were confirmed by flow cytometry experiments. Just 24 h after the addition of **S1**-CPT (200 μg mL^−1^) 50 % of the cells were dead and 25 % had initiated cell death processes. By contrast, no significant reduction in cell viability was observed when cells were treated with **S1**-E, an **S1** solid with no cargo molecule (see the Supporting Information for further details).

**Figure 4:**
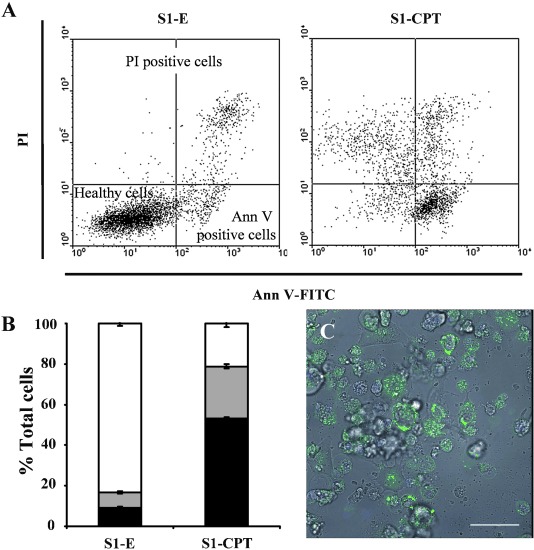
Cell death induction by S1-CPT: A), B) HeLa cells were treated with 200 μg mL^−1^ of S1-CPT or S1-E (S1 without cargo) for 24 h and then flow cytometry studies and quantification of viable (white) and dead cells was performed by PI (black) and Ann V (grey) staining. Two independent experiments containing duplicates were performed. Statistically significant differences were observed (*P*<0.05, Student's t-test). C) Cellular internalisation and release of the cargo of S1-CPT was followed by confocal microscopy (blue) in the presence of the plasma membrane marker WGA-AlexaFluor 647 (green); scale bar: 50 μm.

In summary, we have reported here the synthesis of new nanoscopic silica mesoporous supports capped with enzyme hydrolysable groups for the design of nanodevices for zero release that are specifically opened in the presence of defined enzymes. In particular we have designed gated materials capped with bulky organic moieties containing amide and urea linkages that could be selectively hydrolysed in the presence of amidase and urease, respectively. A remarkably distinct delivery profile was observed depending on the enzyme used. Amidase induced the hydrolysis of two amide bonds located far away from the inorganic support; this allowed immediate, yet incomplete, release of the dye. In contrast, urease hydrolysed the urea bond located deeper inside the capping molecule and closer to the surface of the silica nanoparticle; this allowed a near total cargo release but was delayed in time. Simultaneous treatment with both enzymes displayed a synergistic effect and a delivery profile showing fast and complete payload release was observed. These results demonstrate that it is possible to use relatively simple molecules containing enzyme-hydrolysable groups for the design of versatile capped materials that can be opened at will.

The possibility of including, in the capping molecule, different enzyme-hydrolysable groups located in predefined positions allows the control of the delivery profiles. Based on the fact that enzyme–substrate pairs offer a vast range of combinations, proof-of-concept of the possible application of this nanodevice as drug carrier was performed and proved the ability of the **S1**-CPT solid to be internalized by cells and release its cargo. We believe that the design of multi-enzyme-responsive capped materials can be important in the design of custom-made systems for delivery applications with the aim of controlling the flow of key biological processes in nano- and regenerative medicine.

## Supporting Information

Detailed facts of importance to specialist readers are published as ”Supporting Information”. Such documents are peer-reviewed, but not copy-edited or typeset. They are made available as submitted by the authors.
