# Supplementary material for: Dual Enzyme-Triggered Controlled Release on Capped Nanometric Silica Mesoporous Supports
Source: ChemistryOpen. 2012 Feb 17;1(1):17–20. doi: 10.1002/open.201200003 (PMC3922435; doi:10.1002/open.201200003)
Supplement: Supplementary file 1 [file open0001-0017-SD1.pdf]

ChemistryOPEN

Supporting Information

© Copyright Wiley-VCH Verlag GmbH & Co. KGaA, 69451 Weinheim, 2012

Dual Enzyme-Triggered Controlled Release on Capped Nanometric Silica Mesoporous Supports

Alessandro Agostini,[a, b, c] Laura Mondragón,[a, b, c] Carmen Coll,[a, b, c] Elena Aznar,[a, b, c] M. Dolores Marcos,[a, b, c] Ramón Martínez-Máñez,*[a, b, c] Félix Sancenón,[a, b, c] Juan Soto,[a, b] Enrique Pérez-Payá,[d] and Pedro Amorós[e]

open_201200003_sm_miscellaneous_information.pdf

Chemicals

All of the reactants employed were purchased from Sigma-Aldrich Química S. A. (Madrid, Spain) and used without further purification. For cell biology studies, D-MEM with L-glutamine, fetal calf serum (FCS), trypan blue solution (0.4%) cell culture grade, trypsin, wheat germ agglutinin Alexa Fluor 647, and Hoechst 33342 were provided by Gibco-Invitrogen. The cell proliferation reagent WST-1 was obtained from Roche Applied Science. Camptothecin was provided by Sequoia Research Products, Ltd. Annexin V and propidium iodide were provided by BD Pharmingen.

General Techniques

Powder X-ray diffraction, TG Analysis, elemental analysis, EDX microscopy, N_2 adsorption-desorption techniques were employed to characterize the prepared materials. Powder X-ray measurements were performed on a Philips D8 Advance diffractometer using Cu Kα radiation. Thermo-gravimetric analysis were carried out on a TGA/SDTA 851e Mettler Toledo balance, using an oxidant atmosphere (air, 80 mL/min) with a heating program consisting on a heating ramp of 10 ºC per minute from 393 to 1273 K and an isothermal heating step at this temperature during 30 minutes. TEM images were obtained with a 100 kV Philips CM10 microscope. N_2 adsorption-desorption isotherms were recorded on a Micromeritics ASAP2010 automated sorption analyser. The samples were degassed at 120 ºC in vacuum overnight. The specific surface areas were calculated from the adsorption data in the low pressures range using the BET model. Pore size was determined following the BJH method. Fluorescence spectroscopy was carried out on a Felix 32 Analysis Version 1.2 (Build 56) PTI (Photon Technology International. ^{1}H and ^{13}C nuclear magnetic resonance (NMR) were acquired with Varian 300 spectrometer (Sunnyvale, CA, USA). Live cellular internalization studies were performed with a Cytomics FC 500 (Beckman Coulter Inc.) and a confocal Leica microscope handled with a TCS SP2 system, equipped with an acoustic optical beam splitter (AOBS). Cell viability measurements were carried out with a Wallac 1420 workstation.

Synthesis of the silica mesoporous nanoparticles support (MCM)

The MCM-41 mesoporous nanoparticles were synthesized by the following procedure: *n*-cetyltrimethylammoniumbromide (CTABr, 1.00 g, 2.74 mmol) was first dissolved in 480 mL of deionized water. Then 3.5 mL of NaOH 2.00 M in deionized water was added to the CTABr solution, followed by adjusting the solution temperature to 80ºC. TEOS (5.00 mL, $2.57.10^{-2}$ mol) was then added dropwise to the surfactant solution. The mixture was stirred during 2 h to give a white precipitate. Finally the solid product was centrifuged, washed with deionized water and ethanol, and dried at 60ºC (MCM-41 as-synthesized). To prepare the final porous material (MCM-41), the as-synthesized solid was calcined at 550 ºC using an oxidant atmosphere for 5 h in order to remove the template phase.

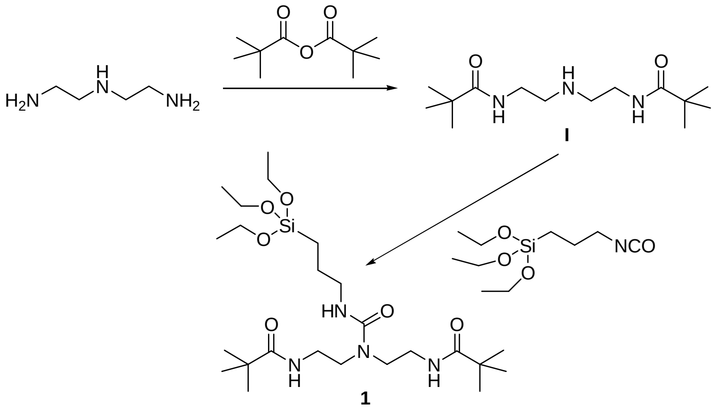

Figure SI-1. Synthesis of compounds **I** and **1**.

Synthesis of product I

A solution of pivaloyl anhydride (1.81 g, 9.72 mmol) in $CHCl_3$ (30 ml) was added dropwise to a chloroform solution (30 mL) of diethylentriamine (0.50 g, 4.8 mmol) at 0ºC during 30 minutes. After this, the reaction mixture was stirred for another 60 miutes and then filtered. The filtrate was extrated with sat. Na_2CO_3 (2 x 20 ml). The $CHCl_3$ layer was then dried (Na_2SO_4), filtered and the solvent removed by rotary evaporation. The resulting white solid (**I**, 0.85 g, 64 % yield) was washed with *n*-hexane. The ^{1}H NMR and ^{13}C NMR data were coincident with the published by Goldcamp et al (see Figure SI-1).[1]

Synthesis of product 1

[1] M. J. Goldcamp, D. T. Rosa, N. A. Landers, S. M. Mandel, J. A. Krause Bauer, M. J. Baldwin, *Synthesis*, **2000**, 2033-2038.

I (900 mg, 3.31 mmol) was dissolved in chloroform (50 mL) and an excess of anhydrous potassium carbonate was added to the solution. Then 3-(triethoxyehtylsilyl)propyl isocyanate (864 mg, 3.50 mmol) was added to the previous mixture in an argon atmosphere. The resultant suspension was stirred at room temperature overnight. The crude reaction was filtered and the solvent removed by rotary evaporation to give the final product (**1**, 1.54 g, 2.98 mmol, 90% yield).
^{1}H RMN (300Mhz, $CDCl_3$): δ 0.83 (m, 2H, CH_2-Si), δ 1.09 (s, 18H, 2(**CH_3**)$_3$-C), δ 1.11 (t, 9H, 3**CH_3**-CH_2-O, J = 3Hz), δ 1.75-1.85 (m, 2H, CH_2-**CH_2**-CH_2), 3.07-3.19 (m, 10H, 5CH_2-NH-CO), 3.72-3.75 (q, 6H, CH_3-**CH_2**-O, J = 3Hz) ppm. ^{13}C δ: 7.78, 18.50, 23.70, 25.34, 27.71, 38.74, 39.86, 44.08, 45.59, 46.34, 58.29, 58.55, 58.68, 159.55, 180.21.

Synthesis of solid S1

With the aim to obtain solid **S1**, MCM-41 calcined (512 mg) was suspended in anhydrous CH_3CN (25 mL) and $[Ru(Bipy)_3]Cl_2 \cdot 6H_2O$ (302 mg, 0.404 mmol) was added. Then 10 ml of CH_3CN were distilled, with a dean-stark from reaction mixture, in order to remove the water present in the pores of the solid. Afterwards the mixture was stirred at room temperature during 24 hours. Subsequently, product **1** (1.33 g, 2.56 mmol) was added and the mixture was stirred overnight. Then the solid was filtered and washed on the filter paper with distilled water and acetone. Finally, in order to remove all the $[Ru(Bipy)_3]Cl_2 \cdot 6H_2O$ present outside of the pores, the solid was suspended in water and stirred at room temperature during 24 hours. Afterwards the solid was filtered and dried at 38ºC overnight.

Synthesis of solid S1-E

With the aim to obtain solid **S1-E**, MCM-41 calcined (208 mg) was suspended in anhydrous CH_3CN (15 mL). Then 10 ml of CH_3CN were distilled, with a dean-stark from reaction mixture, in order to remove the water present in the pores of the solid. Subsequently, product **1** (0.45 g, 0.87 mmol) was added and the mixture was stirred overnight. Then the solid was filtered and washed on the filter paper with distilled water and acetone and dried at 38ºC overnight.

Synthesis of solid S1-CPT

With the aim to obtain solid **S1-CPT**, MCM-41 calcined (600 mg) was suspended in anhydrous CH_3CN (25 mL) and camptothecin (540 mg, 1.5 mmol) was added. Then 10 ml of CH_3CN were distilled, with a dean-stark from reaction mixture, in order to remove the water present in the pores of the solid. Afterwards the mixture was stirred at room temperature during 24 hours. Subsequently, product **1** (1.24 g, 2.4 mmol) was added and the mixture was stirred overnight. Then the solid was filtered and washed on the filter paper with distilled water and acetone. Finally, in order to remove all the camptothecin present outside of the pores, the solid was suspended in water and stirred at room temperature during 24 hours. Afterwards the solid was filtered and dried at 38ºC overnight.

Materials Characterization

Solid **S1** was characterized using standard procedures. Figure SI-2 shows powder X-ray patterns of the nanoparticulated MCM-41 support and the **S1** functionalised material. The PXRD of siliceous nanoparticulated MCM-41 as-synthesized (curve a) shows four low-angle reflections typical of a hexagonal array that can be indexed as (100), (110), (200), and (210) Bragg peaks. A significant displacement of the (100) peak in the XRD powder of the nanoparticulated MCM-41 calcined sample is clearly appreciated in the curve b, corresponding to an approximate cell contraction of 4 Å. This displacement and the broadening of the (110) and (200) peaks are related to further condensation of silanol groups during the calcination step. Finally, curve c corresponds to the **S1** PXRD pattern. In this case, a slight intensity decrease and the disappearance of the (110) and (200) reflections is observed, most likely related to a loss of contrast due to the filling of the pore voids with the ruthenium (II) dye. Nevertheless, the value and intensity of the (100) peak in this pattern strongly evidences that the loading process with the dye and the further functionalization with **1** have not damaged the mesoporous 3D MCM-41 scaffolding.

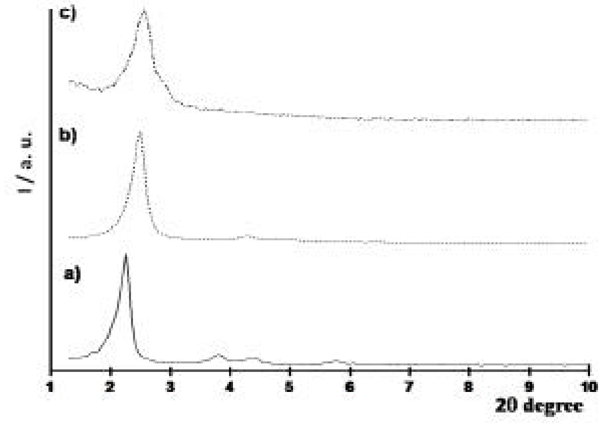

Figure SI-2. Powder X ray pattern of: a) MCM-41 as synthesized; (b) calcined MCM-41 and c) **S1**.

The presence in the final functionalized solid (**S1**) of the mesoporous structure is also confirmed from the TEM analysis, in which the typical channels of the MCM-41 matrix are visualized as alternate black and white stripes (see Figure S1-3 for solid **S1**). The figure also shows that the prepared **S1** are obtained as spherical particles (shape confirmed by SEM image, see figure SI-4) with diameters of ca. 100 nm and. PXRD, TEM and SEM measurements indicated that the loading and grafting procedures did not modify the mesoporous structure of the starting material.

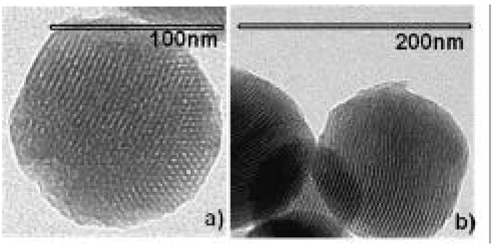

Figure SI-3. TEM image of a) the MCM-41 solid support and b) solid **S1**, showing the typical hexagonal porosity of the MCM-41 matrix.

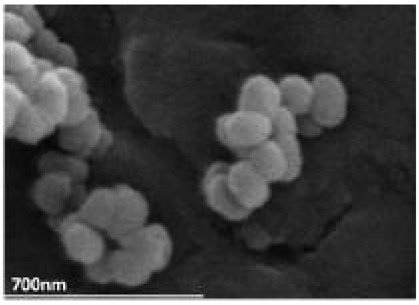

Figure SI-4. SEM image oft he solid **S1** showing the typical spherical shape of the nanoparticles.

The N_2 adsorption-desorption isotherms of the nanoparticulated MCM-41 calcined material shows an adsorption step at intermediate P/P_0 value (0.1-0.4) typical of this solids (see Figure SI-5). This step can be related to the nitrogen condensation inside the mesopores by capillarity. The absence of a hysteresis loop in this interval and the narrow BJH pore distribution suggest the existence of uniform cylindrical mesopores with pore volume of 1.18 $cm^3\ g^{-1}$ calculated by using the BJH model on the adsorption branch of the isotherm. The application of the BET model resulted in a value for the total specific surface of 999.6 m^2/g and a pore volume of 0.79 $cm^3\ g^{-1}$. From the XRD, porosimetry and TEM studies, the a_0 cell parameter (4.44 nm), the pore diameter (2.46 nm) and a value for the wall thickness (1.99 nm) were calculated. In addition to this adsorption step associated to the micelle generated mesopores, a second feature appears in the isotherm at a high relative pressure ($P/P_0 > 0.6$). This adsorption correspond to the filling of the large voids among the particles and present a volume of 0.30 $cm^3\ g^{-1}$ (calculated by using the BJH model) and then must be considered as a textural-like porosity. In this case, the curves show a characteristic H1 hysteresis loop and a wide pore size distribution.

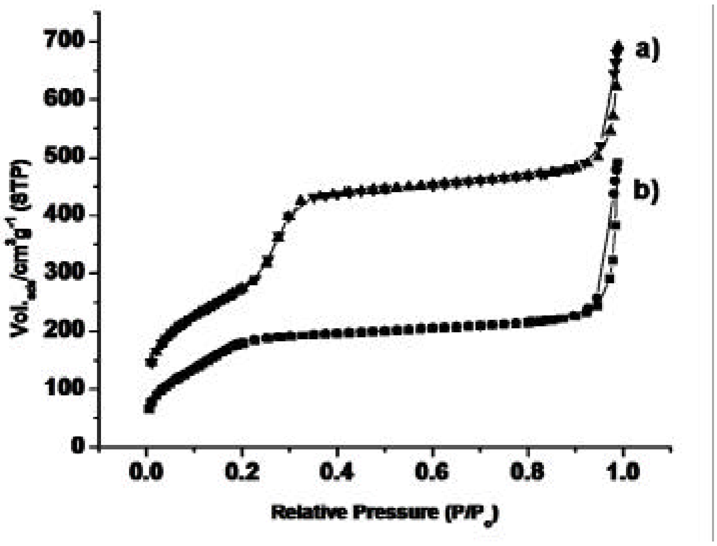

Figure SI-5. Nitrogen adsorption-desorption isotherms for a) MCM-41 mesoporous material and b) **S1** material.

The N_2 adsorption-desorption isotherm of **S1** is typical of mesoporous systems with filled mesopores (see Figure SI-5), and a significant decrease in the N_2 volume adsorbed and surface area (696.5 m^2/g) is observed. Additionally, a certain textural porosity is preserved. BET specific surface values, pore volumes, and pore sizes calculated from the N_2 adsorption-desorption isotherms for MCM-41 and **S1** are listed in Table 1.

Table 1. BET specific surface values, pore volumes and pore sizes calculated from the N_2 adsorption-desorption isotherms for selected materials.

	S_{BET} (m^2 g^{-1})	**Pore Volume** [a] (cm^3 g^{-1})	**Pore size** [a] (nm)
**Mesostructured MCM-41**	64.4	0.21	-
**MCM-41**	999.6	0.79	2.46
**S1**	696.5	0.41	2.14

[a] Volume (V) and diameter (D) of mesopore.

The content of product **1**, ruthenium (II) dye and camptothecin in solids **S1**, **S1-E** and **S1-CPT** was determined by thermogravimetric analysis. Values of contents are detailed in Table 2.

Table 2. Content of product **1**, complex $Ru(bipy)_3^{2+}$ and camptothecin in the prepared solids **S1**, **S1-E** and **S1-CPT** in mmol/g SiO_2.

**Solid**	**1**	**$Ru(bipy)_3^{2+}$**	**Camptothecin**
**S1**	0.15	0.22	-
**S1-E**	0.13	-	-
**S1-CPT**	0.61	-	0.43

Delivery studies of solid S1

A typical experiment was carried out in batch and four different suspensions were prepared: (a) 5 mg of **S1** and 12.5 ml of water at pH = 7.5 (blank); (b) 5 mg of **S1**, 12.5 ml of water at pH = 7.5 and 20 μl of urease from *Canavalia ensiformis*; (c) 5 mg of **S1**, 12.5 ml of water at pH = 7.5 and 1 μl of amidase from *Pseudomonas aeruginosa*; (d) 5 mg of **S1**, 12.5 ml of water at pH = 7.5 and 1 μl of amidase from *Pseudomonas aeruginosa*. The results obtained are showed in Figure SI-6.

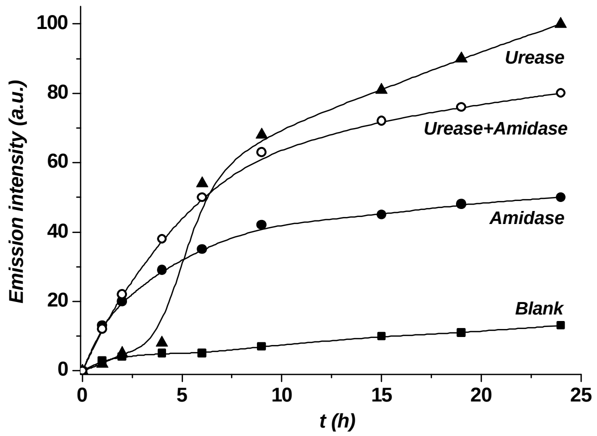

Figure SI-6. Kinetics of the release of $[Ru(bipy)_3]^{2+}$ dye from solid **S1** in the presence of amidase and urease enzymes.

Cell Culture Conditions

The HeLa human cervix adenocarcinoma and the MCF-7 breast cancer cells were purchased from the German Resource Centre for Biological Materials (DSMZ) and were grown in D-MEM supplemented with 10% of FCS. Cells were maintained at 37 °C in an atmosphere of 5% carbon dioxide and 95% air and underwent passage twice a week.

WST-1 Cell Viability Assay

Cells were cultured in sterile 96-well microtiter plates at a seeding density of 2500 and 3000 cells/well for HeLa and MCF-7, respectively, and they were allowed to settle for 24 h. **S1** in DMSO was added to cells at a final concentration of 200, 100 and 50 µg/mL. After 23 hours, WST-198 (7 µL of a 5 mg/mL solution) was added to each well. Cells were further incubated for 1 h (a total of 24 hours of incubation was therefore studied), and absorbance was measured at 450 nm. The results obtained are depicted in Figure SI-7. No cell toxicity associated to nanoparticles was observed in HeLa or MCF-7 cells.

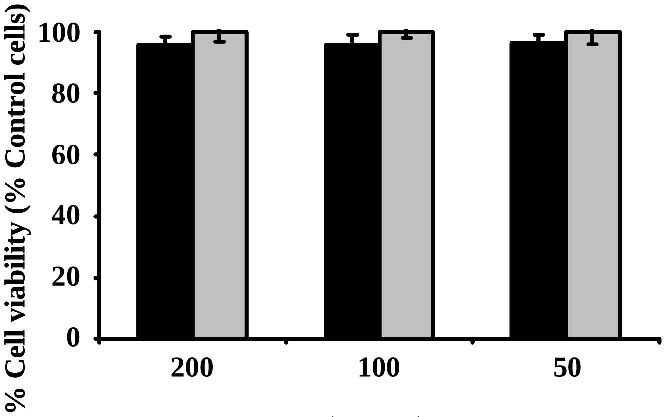

Figure SI-7. HeLa (black) and MCF-7 (grey) cells were incubated for 24 hours with **S1** at concentrations of 200, 100 and 50 µg/mL and cell viability was quantified employing WST-1 reagent. Two independent experiments with quadruplicates were performed. Data are expressed as (mean ± SE).

Live Confocal Microscopy S1 Cellular Internalization

HeLa and MCF-7 cells were seeded in 24 mm ϕ glass coverslips in six-well plates at a seeding density of 100000 or 50000 cells/well for 24 hours or 48 hours incubation assays, respectively. After 24 hours, cells were treated when indicated with **S1**, **S1-CPT** or **S1-E** at concentrations of 200 and 100 µg/mL. Then, cells were incubated for 24 or 48 hours prior confocal microscopy studies. For this purpose, cells were stained when indicated with 10 ng/mL of Hoechst 33342 and 5 mg/mL wheat germ agglutinin (WGA) Alexa Fluor 647 for 30 minutes in PBS containing 10% FCS or keeping the medium in case of **S1-CPT** and **S1-E** treatments. Slides were visualized under a confocal microscope.

The internalization of **S1** nanoparticles can be observed in Figure SI-8 by means of $[Ru(bipy)_3]^{2+}$-associated fluorescence (red). A cytoplasmic vesicular signal can be observed in both cell lines proving the internalization of the nanoparticles.

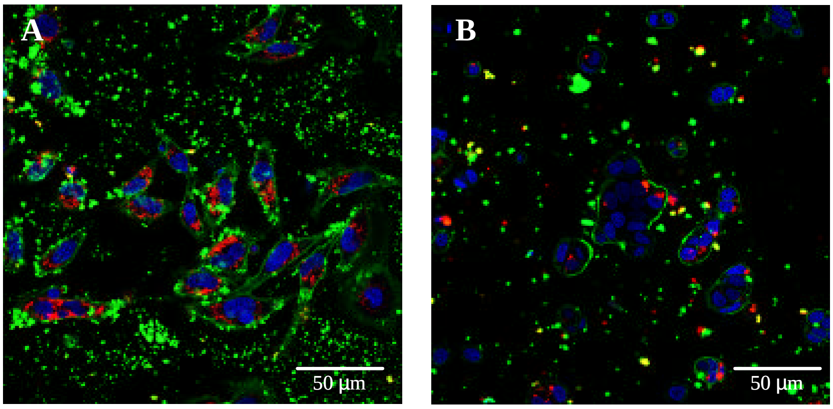

Figure SI-8. **S1** cellular internalization. HeLa (A) and MCF-7 (B) cells were treated with **S1** at concentration of 100 µg/mL for 24 hours. Then, confocal microscopy studies of **S1** cellular uptake were performed by means of $[Ru(bipy)_3]^2$-**S1** associated fluorescence (red) in the presence of the DNA marker Hoechst 33342 (blue) and the plasma membrane marker WGA Alexa Fluor 647 (green). Two independent experiments were performed obtaining similar results.

Cytofluorometry Studies Employing S1

To develop the cytofluorometry studies, HeLa and MCF-7 cells were seeded at 12500 or 25000 cells/well in a 24-well in case of HeLa or 20000 or 40000 cells/well in case of MCF-7 for 24 and 48 hours incubation assays, respectively. plate. After 24 hours, cells were treated with **S1-CPT** or **S1-E** at concentrations of 200 and 100 µg/mL. Cells were incubated for 24 or 48 hours prior staining them with PI and Ann V according to manufacturer's protocol (BD Pharmingen). Quantification of PI positive and Ann V positive stainings was performed employing WinMDI program version 2.9 (see Figure SI-9).

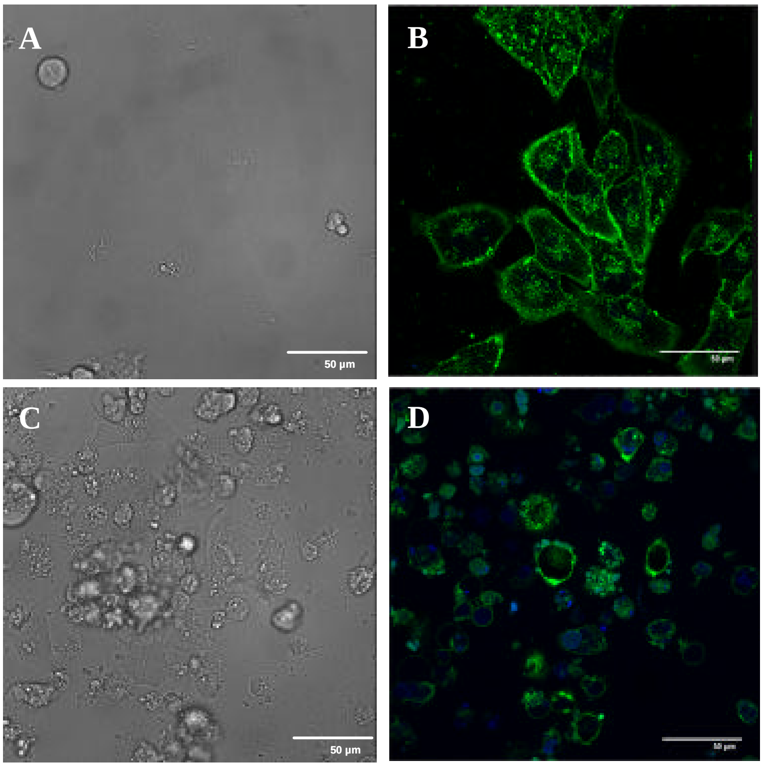

Figure SI-9. HeLa cells were treated with 200 µg/mL of **S1-E** (A, B) or **S1-CPT** (C, D) for 24 hours. Then, cellular internalization and release of the cargo of **S1-CPT** was followed by confocal microscopy (blue) in the presence of the plasma membrane marker WGA Alexa Fluor 647 (green). Experiment was performed twice with similar results.
